# Effect of a Physical Exercise Program on the Inflammatory Response, Cardiac Functions, Functional Capacity, and Quality of Life in Patients with Sickle Cell Disease

**DOI:** 10.3390/jcm12123952

**Published:** 2023-06-09

**Authors:** Daniele Andreza Antonelli Rossi, Jonas Alves De Araujo Junior, Gustavo José Luvizutto, Rodrigo Bazan, Péricles Sidnei Salmazo, Gabriel Pinheiro Modolo, João Carlos Hueb, Hélio Rubens de Carvalho Nunes, Newton Key Hokama, Marcos Ferreira Minicucci, Meliza Goi Roscani, Silméia Garcia Zanati Bazan

**Affiliations:** 1 Department of Internal Medicine, Botucatu Medical School-UNESP, São Paulo State University, Botucatu 18618-687, Brazil; danisaps@yahoo.com.br (D.A.A.R.); xonasxr@gmail.com (J.A.D.A.J.); jchueb@uol.com.br (J.C.H.); newton.hokama@unesp.br (N.K.H.); marcos.minicucci@unesp.br (M.F.M.); 2 Department of Applied Physical Therapy, Federal University of Triângulo Mineiro, Uberaba 38065-430, Brazil; gluvizutto@gmail.com; 3 Department of Neurology, Botucatu Medical School-UNESP, São Paulo State University, Botucatu 18618-970, Brazil; bazan.r@terra.com.br (R.B.); g.modolo@unesp.br (G.P.M.); 4 Department of Medicine, Faculty of Medical and Health Sciences, Pontifical Catholic University of São Paulo, Sorocaba 18052-490, Brazil; pesalmazo@yahoo.com.br; 5 Department of Biostatistics, Botucatu Medical School-UNESP, São Paulo State University, Botucatu 18618-689, Brazil; hrcn@outlook.com.br; 6 Department of Medicine, Federal University of Sao Carlos-UFSCar, São Carlos 13565-251, Brazil; meliza.roscani@gmail.com

**Keywords:** hemoglobinopathies, functional capacity, physical activity, inflammation

## Abstract

Introduction: The beneficial effects of physical exercise on functional capacity and inflammatory response are well-known in cardiovascular diseases; however, studies on sickle cell disease (SCD) are limited. It was hypothesized that physical exercise may exert a favorable effect on the inflammatory response of SCD patients, contributing to an improved quality of life. This study aimed to evaluate the effect of a regular physical exercise program on the anti-inflammatory responses in SCD patients. Methods: A non-randomized clinical trial was conducted in adult SCD patients. The patients were divided into two groups: 1—Exercise Group, which received a physical exercise program three times a week for 8 weeks, and; 2—Control Group, with routine physical activities. All patients underwent the following procedures initially and after eight weeks of protocol: clinical evaluation, physical evaluation, laboratory evaluation, quality of life evaluation, and echocardiographic evaluation. Statistical analysis: Comparisons between groups were made using Student’s *t*-test, Mann–Whitney test, chi-square test, or Fisher’s exact test. Spearman’s correlation coefficient was calculated. The significance level was set at *p* < 0.05. Results: There was no significant difference in inflammatory response between the Control and Exercise Groups. The Exercise Group showed an improvement in peak VO_2_ values (*p* < 0.001), an increase in the distance walked (*p* < 0.001), an improvement in the limitation domain due to the physical aspects of the 36-Item Short Form Health Survey (SF-36) quality of life questionnaire (*p* = 0.022), and an increase in physical activity related to leisure (*p* < 0.001) and walking (*p* = 0.024) in the International Physical Activity Questionnaire (IPAQ). There was a negative correlation between IL-6 values and distance walked on the treadmill (correlation coefficient −0.444, *p* = 0.020) and the estimated peak VO_2_ values (correlation coefficient −0.480; *p* = 0.013) in SCD patients in both groups. Conclusions: The aerobic exercise program did not change the inflammatory response profile of SCD patients, nor did it show unfavorable effects on the parameters evaluated, and patients with lower functional capacity were those with the highest levels of IL-6.

## 1. Introduction

SCD is an autosomal codominant disease caused by abnormal beta-globin alleles that carry a sickle cell mutation in the HBB gene. It is one of the most common monogenic disorders in hematology [1]. The pathophysiology of SCD is related to structural alterations that occur in sickle-shaped red blood cells (HbS) due to structural changes in chromosome 11 [2]. SCA has multisystemic clinical manifestations such as chronic microvascular occlusion, reduced blood flow to vital organs, and immune system changes that can occur from childhood to adulthood [3,4,5,6].

Hemolytic anemia, vaso-occlusion, and inflammation are the main hallmarks of the chronic complications of these patients [7]. Other complications, such as endothelial dysfunction, pulmonary hypertension, renal dysfunction, and stroke, seem to have hemolytic anemia as the main trigger, increasing exercise intolerance and risk of heart failure and representing an independent risk factor for mortality [8,9,10,11,12]. Regarding vaso-occlusive crises, their mechanism is complex, multifactorial, and not yet fully understood. The adhesion of erythrocytes and leukocytes to the endothelium and the presence of vasoactive mediators and inflammatory cytokines seem to play important roles in the disease pathophysiology [13].

SCD causes chronic inflammation with increased pro-inflammatory mediators and circulating polymorphonuclear cells [14,15]. Pro-inflammatory cytokines, such as IL-1, IL-6, and TNF-α, cause chronic endothelial activation and play a role in tissue ischemia and local necrosis [16]. Elevated levels of TNF-α are related to severe disease and the presence of cell adhesion molecules. Evidence suggests that a succession of interrelated and cyclic events accounts for the mechanisms involved in the genesis of vaso-occlusion [17,18]. A source of concern regarding the practice of exercise in SCD patients is the consequent increase in muscle oxygen consumption and the release of oxygen by red blood cells, which increases the chance of HbS polymerization [19].

Studies also indicate an increased risk of red blood cell deformation and vaso-occlusion with exercise intensity [20]. However, the habit of exercising regularly in the general population leads to long-term cardiovascular adaptations and promotes aerobic conditioning through organic structural and functional changes [21]. Favorable effects on maximal oxygen consumption (VO_2_ max) have been described in healthy, young, and elderly patients after relatively short periods of aerobic training at 70% of VO_2_ max; sessions lasting 45 min three times a week were sufficient to promote improvement from the third week onwards, with additional increments up to the twelfth week [22].

In SCA patients, studies with exercise protocols at moderate intensity and volume demonstrated positive effects on ventilatory efficiency and reduction in oxidative stress after a training period lasting 6 weeks [23]. There is also evidence of improvement in muscle capillarization after aerobic training for 8 weeks [24]. Several studies have also shown that regular physical activity is associated with chronic anti-inflammatory effects in healthy individuals [25]. Similar findings have been reported in cardiovascular and autoimmune diseases [26,27,28]. Exercise can promote the production of IL-10, a cytokine with anti-inflammatory effects, and reduce TNF-α levels [29]. Thus, regular physical activity has been investigated to improve the health status of individuals with sickle cell disease, as it has been proven in other cardiovascular, metabolic, and respiratory diseases [20].

Currently, intense exercise is considered inappropriate for sickle cell disease and is a potential cause of painful crises. However, light to moderate physical activity under adequate environmental conditions and good hydration status seems well tolerated and presents evidence of safety in SCD patients [19]. Exercise intolerance contributes to difficulties in daily living activities, leading to physical, psychological, and social damage. Therefore, this study aimed to analyze the effect of a physical exercise program on the inflammatory markers, morphofunctional echocardiographic variables, functional capacity, physical activity level, and quality of life in SCD patients, and correlate functional capacity variables with inflammatory responses. The main hypothesis of the study is that regular physical exercise can have a favorable effect on the inflammatory markers, contributing to the improvement in the functional capacity and quality of life in SCD patients.

## 2. Methods

### 2.1. Study Design, Setting, and Participants

A prospective, non-randomized, unicentric longitudinal clinical trial was conducted involving patients diagnosed with SCD between 2015 and 2017. Patients were referred from the hemoglobinopathies outpatient clinic of the Botucatu Medical School, UNESP. All patients who had periodic consultations at the service were invited to participate in the project, taking advantage of the moment they waited for routine consultation at the Medical Service. The invitation to participate in the study was carried out by the service physician, together with the physical education teacher responsible for guiding and monitoring the execution of the exercise protocol. The study protocol was approved by the Research Ethics Committee of the Botucatu Medical School (20612913.5.0000.5411) and registered in the Brazilian Registry of Clinical Trials (ReBEC-RBR-29X8QK). The patients were informed about the procedures and signed an informed consent form.

Patients of both sexes diagnosed with SCD (SS or SC pattern), aged over 18 years, were included. Individuals who had a painful crisis in the past 30 days at the time of inclusion, patients with recurrent infections, or patients with daily painful crises that limited physical exercise were excluded. After inclusion, patients with serious adverse effects during the execution of the exercise protocol, non-adaptation to the protocol, or non-adherence to exercise were excluded.

### 2.2. Interventions

The volunteers were allocated to two groups based on their voluntary expression of interest in participating in the exercise program.

#### 2.2.1. Exercise Group

This was composed of patients who performed a program of regular physical exercise for 8 weeks and a frequency of 3 times a week, lasting 1 h. The exercise protocol was based on low-intensity aerobic activities with increasing duration, allowing moderate intensity as the individuals showed improvement in physical conditioning. The exercise protocol consisted of three moments:

(a) Initial phase: calisthenics and flexibility exercises, lasting approximately 10 min.
(b) Main phase: aerobic exercise, with walking guidance and gradual increase in intensity and duration, with prescription as follows: In the first two weeks: 35 min of walking between 60% and 70% of maximum heart rate (HR max) achieved in the treadmill test; third and fourth week: 40 min of walking between 60% and 70% of HR max; fifth and sixth week: 40 min of walking between 65% and 75% of HR max; seventh and eighth week: 50 min of walking between 65% and 75% of HR max.
(c) Final phase: relaxation phase, 10–15 min of calisthenics and flexibility exercises. This exercise protocol was based on previous studies and current recommendations that advocate low-intensity activities for individuals with sickle cell anemia, as high-intensity exercises can trigger painful crises. Current recommendations for maintaining health in the general population include moderate-intensity aerobic exercise, 150 min/week, but emphasize that sedentary individuals can benefit from lower volumes and intensities and with individualized progression [30,31]. The physical education teacher monitored activities carried out weekly via telephone.

#### 2.2.2. Control Group

No prescription of physical exercise but instructed to maintain their daily activities for 8 weeks.

## 3. Outcomes

### 3.1. Primary

Inter-group differences (delta M2-M1) in circulating levels of cytokines (TNF-a, IL-1, IL-6, IL-10, PCR, and BNP) expressed in picograms per milliliter (pg/mL).

### 3.2. Secondary

Inter-group differences (delta M2-M1) in morphofunctional echocardiographic variables measured by echocardiography, functional capacity measured by an ergometric test, physical activity level measured by IPAQ, and quality of life measured by the Medical Outcomes Study (SF-36).

## 4. Measurements

All patients were evaluated at baseline (M1) and after 8 weeks of intervention (M2). Assessments were performed by an examiner blinded to the patient allocation group (Exercise or Control) using the following procedures:

### 4.1. Clinical Evaluation

The clinical evaluation was performed by a hematologist and consisted of a clinical record, general and special physical examination, medications in use, and treatments performed.

### 4.2. Anthropometric Assessment

The anthropometric assessment consisted of body weight (BW) and height (H) measurements to determine the Body Mass Index (BMI). To measure body weight, a platform-type scale (Filizola) was used with a maximum capacity of 150 kg and a precision of 0.1 kg, and at the time of weighing, patients were wearing light clothes and without shoes. A portable stadiometer with an accuracy of 0.1 cm was used for the height measurement, considering the arithmetic mean of three consecutive measurements as the final result. BMI was calculated using the following formula: BMI = BW (kg)/[H (m)]^2^. Overweight was diagnosed when the BMI was between 25 and 30 kg/m^2^, whereas obesity was diagnosed when the BMI was equal to or greater than 30 kg/m^2^ [32].

### 4.3. Analysis of Inflammatory Biomarkers

For both groups, two samples of venous blood (10 mL) were collected in tubes containing ethylenediaminetetraacetic acid (EDTA) (Vacutainer^®^, Becton Dickinson, Franklin Lakes, NJ, USA). The blood collection procedure was performed in the hematology department using appropriate disposable materials by a trained and qualified professional. Plasma was separated by centrifugation and stored at −80 °C.

Plasma levels of IL-12p70, IL-1b, IL-8, IL17, IFN-g, TNF-a, IL10, IL-6, IL-4, and IL-2 were assessed using a cytometric bead array (BD Bioscience, San Jose, CA, USA) according to the manufacturer’s instructions. The levels of these cytokines were used to compare independent and dependent variables. The plasma levels of (TNF-a, IL-1, IL-6, IL-10, CRP, and BNP) were assessed using enzyme-linked immunosorbent assay (ELISA) (Multiskan EFLAB, Helsinki, Finland) according to the manufacturer’s instructions (Wuhan EIAab, Science Co., Ltd., Wuhan, China, cat no. E0541h). Cytokine levels were expressed as pg/mL.

### 4.4. Doppler Echocardiographic Assessment

Doppler echocardiographic examinations were performed by an echocardiography specialist using the Vivid S6 equipment (General Electric Medical Systems, USA), with a 2.0–3.5 MHz multifrequency ultrasonic transducer. The patients were positioned in the left lateral decubitus position, with the left upper limb slightly flexed under the head. The electrocardiographic lead was monitored continuously. Images were obtained and analyzed following the recommendations of the American Society of Echocardiography [33] and the Canadian Consensus for flow analysis [34].

### 4.5. Functional Capacity Assessment—Ergometric Test

To assess functional capacity, an exercise stress test was performed by a cardiologist specialized in a treadmill model Centurion 300, using the ErgoPC13 Micromed^®^ program with 12 classic leads and a modified CM5 lead. The Mini Bruce protocol was used—3-min stages and load increments, as follows: 2.7 km/h with 0% slope; 2.7 km/h with 10% slope; 4.0 km/h with 12% slope; 5.5 km/h with 14% slope; 6.8 km/h with 16% slope; 8 km/h with 18% slope. The calculation of maximum oxygen consumption (estimated peak VO_2_) was performed indirectly using Balke’s equation: VO_2_ = v × (0.073 + ts/100) × 1.8, where ts = treadmill slope in percentage, v = treadmill speed in m/min [35,36,37]. The examinations were performed in an air-conditioned ergometry room to avoid exposing patients to excessive cold or heat, with the care of hydration during and after the examinations.

### 4.6. Degree of Physical Activity Assessment

The IPAQ was used to obtain information about the habitual physical activity of the study participants [38]. After the questionnaire application, the individuals were classified as very active, active, irregularly active (A and B), or sedentary.

### 4.7. Quality of Life Assessment

The Medical Outcomes Study SF-36 is a multidimensional questionnaire comprising 36 items encompassed in 8 scales or dimensions: functional capacity, physical aspects, pain, general health status, vitality, social aspects, emotional aspects, and mental health. The quality of life score ranges from 0 to 100, with values close to 0 considered worse and values close to 100 considered better [39].

### 4.8. Statistical Methods

For parametric variables, the values obtained are presented as mean and standard deviation (SD); for non-parametric variables, the values are expressed as median, 25%, and 75%. The difference between the final (M2) and baseline (M1) time points was calculated for each variable between the groups. The deltas of variables in the Control and Exercise Groups were compared. For continuous variables, comparisons between groups were performed using Student’s *t*-test if the distribution was normal or the Mann–Whitney test if the distribution was non-normal. For categorical variables, the chi-squared or Fisher’s exact test was used. Associations between the clinical and laboratory variables were assessed using Spearman’s correlation coefficient. Data analyses were performed using the SigmaPlot software for Windows v12.0 (Systat Software Inc., San Jose, CA, USA). The significance level was set at *p* < 0.05.

## 5. Results

**Figure 1:**
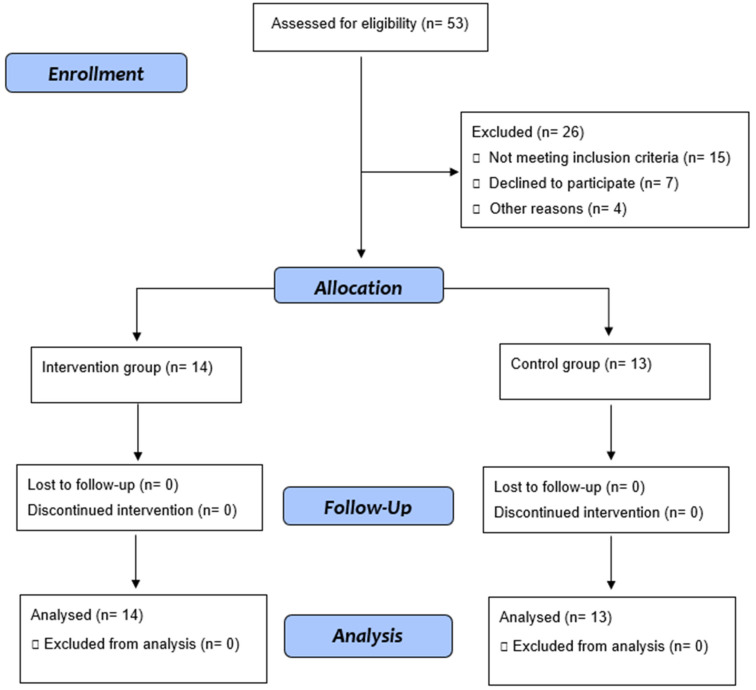
Flow diagram.

Fifty-three adults diagnosed with SCD were selected for this study. Of these, 27 agreed to participate in the study (Figure 1). The analysis of the baseline characteristics of individuals in the Control and Exercise Groups at baseline (M1) did not show significant differences between the groups (Table 1). There was no difference between the groups in M1 and M2 with the values of serum hemoglobin, hematocrit, platelets, or white blood cells (Table 2).

After the exercise program, no significant changes were observed in TNF-α, IL-6, IL-1, IL-10, CRP, and BNP levels between the groups (Table 3). In the echocardiographic evaluation, the Exercise Group showed an increase in the diastolic thickness of the interventricular septum (*p* = 0.027), an increase in the systolic excursion velocity of the tricuspid annulus on tissue Doppler (St wave), a variable related to the systolic function of the right ventricle (*p* = 0.015), and improvement in ejection fraction (*p* = 0.021) compared to the Control Group (Table 4).

Table 5 shows the levels of physical activity, functional capacity, and quality of life in both groups. At the end of the intervention, the physical Exercise Group showed an increase in physical activity related to leisure (*p* < 0.001), walking (*p* = 0.024), and total activity (*p* = 0.021) compared to the Control Group. Regarding functional capacity, the Exercise Group showed a significant difference in terms of time (*p* = 0.005) and distance (*p* < 0.001) covered on the treadmill and the estimated peak VO_2_ (*p* < 0.001) compared to the Control Group. There was also a significant difference in heart rate (HR) assessed at rest before the beginning of the exercise test, with a drop in resting HR values in the group subjected to the exercise program at M2 (*p* = 0.034). In terms of quality of life, there was a significant improvement in the physical aspect limitation domain in the exercise program group (*p* = 0.022) compared to the Control Group.

Table 6 shows the correlation of cytokines with parameters evaluated in the treadmill test (walking time, distance covered on the treadmill, and peak VO_2_) for all SCD patients (Control and Exercise Groups combined). Patients with higher IL-6 values had the shortest distance walked on the treadmill (correlation coefficient, −0.444; *p* = 0.020) and the lowest estimated VO_2_ peak (correlation coefficient, −0.480; *p* = 0.013).

## 6. Discussion

After the exercise program, no significant changes were observed between the groups regarding cytokine dosage. However, it was observed that in the intervention group, there was an increase in right ventricular systolic function and left ventricular ejection fraction and increased physical activity, functional capacity, and quality of life compared to the Control Group.

Studies have shown that serum cytokine levels in SCD patients are elevated even in the “steady state” [40,41]. Elevation of IL-6 in this steady state between crises has also been associated with impaired immune response and increased morbidity. In a vaso-occlusive crisis, increased levels of IL-6 and other cytokines are even more evident [42]. Regular physical activity is associated with anti-inflammatory effects and is related to improved deformability of red blood cells, reduction in blood viscosity, and adaptations for cell aggregation. However, no significant differences in cytokine levels were observed between the groups. In contrast, Abd El-Kader et al. observed a reduction in TNF-α and IL-6 levels after 12 weeks of aerobic training [43]. Studies on the effect of exercise in healthy sedentary individuals showed that the decrease in IL-6 and CRP occurred only after long intervention periods and with higher exercise intensities [44].

Despite the absence of improvement in serum cytokine levels, it is noteworthy that the exercise protocol performed was not characterized as an aggressor factor that could worsen the inflammatory pattern, which is important because this mechanism is involved in painful crises and their consequences. In our study, we observed higher levels of IL-6 in SCD patients who had lower functional capacity. The increase in circulating IL-6 was previously associated with several chronic diseases, and its plasma concentration is related to the rise in general mortality and cardiovascular causes [29]. Regular physical exercise seems to influence the expression of IL-6, and lower plasma concentrations of these cytokines at rest and in response to exercise are characteristic of adaptation to training [25]. Thus, it seems consistent with the data found that study patients with lower physical fitness have the highest levels of circulating IL-6. Additionally, it is believed that longer protocols can reduce the inflammatory profile of this population.

In the analysis of echocardiographic data, no significant differences were found in the morphological variables. However, there was an improvement in the systolic function of the Exercise Group at the end of the protocol. As a hypothesis, the improvement in ejection fraction may be related to the reduction in afterload due to the improvement in endothelial function, as suggested in patients with heart failure with reduced ejection fraction [45]. Increased nitric oxide production as an effect of regular practice of physical activity has been described in patients with heart failure [46]. Increased nitric oxide production or greater availability has been reported in SCA patients after moderate-intensity exercise protocols [47].

In addition, this study observed an improvement in the estimated peak VO_2_ values and an increase in the time and distance covered by patients, reflecting an increase in functional capacity and improved tolerance to the effort, which is one of the major limitations of daily activities in individuals with SCD. Although there was no improvement in the functional capacity domain of the SF-36 questionnaire, there was a significant improvement in the physical limitations. In conjunction with the favorable results of the IPAQ in the walking and leisure domains, we can infer that the patients showed improvement in important aspects related to the quality of life after 8 weeks of regular exercise. Studies in animal models have shown a reduction in intramuscular acidosis after endurance training, possibly associated with improved vascular and inflammatory parameters and a reduction in oxidative stress [48].

It should be noted that in both groups, the hemoglobin and hematocrit values were reduced and similar and did not change during the study period, thus eliminating the possible influence of this factor on the results presented. It must be considered that SCD has a variable clinical presentation, which can manifest as sporadic vaso-occlusive crises or significant lesions in different organs. It is influenced by genetic differences and regional and environmental conditions [49]. The wide clinical diversity can play a prominent role in the observed data and act on the heterogeneous results available in the literature regarding the effect and benefit of exercises in SCD patients and with the profile of inflammatory activity. This is an intrinsic characteristic of the disease, making it difficult to reproduce and generalize the results in this population, especially when considering the association of this clinical diversity with the relatively small sample of individuals evaluated, which happens with most studies carried out with SCD patients.

Even considering the possible difficulties of universalization, the intervention proved to be easily applicable because of the low complexity of the protocols adopted. Exercising in a home environment increases the possibility of reaching more people and restricts possible limitations, such as inadequate space or equipment. The initial clinical evaluation described is part of the routine follow-up of these patients in most services. The complementary tests used before participation (echocardiogram and exercise tests) can be included without major difficulties. As long as an SCD patient manifests the desire to perform physical activity, the orientation of regular aerobic exercises of light to moderate intensity can be performed safely for different genotypes, since it has neutral effects on inflammatory activity without worsening the pain or crisis-triggering and can bring beneficial results in terms of tolerance to efforts and improved quality of life. Regarding the limitations of the study, in addition to the fact that it was not possible to develop a randomized study and this was unicentric, the typically low socioeconomic profile of the patients, as demonstrated in studies aimed at this investigation, also created some obstacles to the follow-up [50]. Due to difficulties in commuting between the patients’ city of residence and the study site, it was not possible to carry out the exercise program in a direct supervised manner.

## 7. Conclusions

The aerobic exercise program performed for 8 weeks improved cardiac function, functional capacity, physical activity level, and quality of life without altering the inflammatory response profile of SCD patients. In addition, there were no adverse effects. Patients with lower functional capacity had the highest levels of circulating IL-6, suggesting an increase in basal inflammatory activity. Regular physical activity in light to moderate intensity seems safe and has beneficial effects on effort tolerance and improving quality of life in this population. Phase 3 randomized clinical trials should confirm these findings using a larger sample size.

## Figures and Tables

**Table 1:** Baseline clinical and demographic characteristics of the Control and Exercise Groups.

Variables.	Control Group (*n* = 13)	Exercise Group (*n* = 14)	*p*
Age (years)	25 (22.5–36.5)	27.5 (22.6–36)	1.0
Female	5 (38.5%)	9 (64.3%)	0.339
Weight (kg)	57.6 ± 14.5	60.0 ± 13.3	0.403
SAH, *n* (%)	0	1 (7.1%)	1.0
Obesity, *n* (%)	0	1 (7.1%)	1.0
Dyspnea, *n* (%)	3 (23.1%)	6 (42.9%)	0.42
Folic acid, *n* (%)	13 (100%)	12 (85.7%)	0.48
Hydroxyurea, *n* (%)	3 (23.1%)	5 (35.7%)	0.678

Values are expressed as median and 25th and 75th percentiles, absolute values and percentages, or mean ± standard deviation. SAH: systemic arterial hypertension. Significance level *p* < 0.05.

**Table 2:** Comparison of blood count parameters between the Control and Exercise Groups at moments M1 and M2.

Variables	Control Group (*n* = 13)			Exercise Group (*n* = 14)			*p*
	M1	M2	M2-M1	M1	M2	M2-M1	
Hemoglobin (g/dL)	9.70 (7.50–10.4)	10.2 (8.65–11.0)	0.70 (0.42–1.15)	9.30 (7.95–10.6)	9.90 (9.05–11.1)	0.60 (−0.28–1.53)	0.942
Hematocrit (%)	30.2 (23.5–31.4)	29.1 (23.8–35.0)	0.90 (−1.15–3.50)	30.5 (24.5–36.0)	31.1 (25.6–34.4)	1.00 (−1.60–2.00)	0.645
Platelets (mil/mm^3^)	343 (239–485)	322 (255–453)	4.00 (−43.5–29.0)	493 (388–546)	464 (385–566)	−0.5 (−35.8–37.8)	0.923
White blood cells (cells/mm^3^)	13,300 (8800–20,350)	12,500 (9700–15,350)	0.30 (−1.00–1.05)	13,250 (11,575–17,175)	13,350 (9700–17,025)	0.45 (−0.50–1.60)	0.451

Values are expressed as median and 25th and 75th percentiles at pre (M1) and post (M2) moments in both groups, and as median and 25th and 75th of deltas between moments M2 and M1 in both groups. Significance level *p* < 0.05.

**Table 3:** Comparison of dosages of inflammatory biomarkers between the Control and Exercise Groups at moments M1 and M2.

Variables	Control Group (*n* = 13)			Exercise Group (*n* = 14)			*p*
	M1	M2	M2-M1	M1	M2	M2-M1	
TNF-alpha (pg/mL)	9.87 (9.18–11.1)	10.5 (9.41–10.8)	0.53 (0.16–0.90)	9.60 (8.73–10.6)	10.6 (9.03–11.1)	1.03 (−1.36–2.12)	0.482
IL-6 (pg/mL)	3.81 ± 1.45	3.46 ± 0.55	−0.36 ± 1.23	3.53 ± 0.83	3.09 ± 0.53	−0.44 ± 0.80	0.829
IL-1 (pg/mL)	5.29 ± 0.38	5.23 ± 0.56	−0.06 ± 0.49	5.23 ± 0.51	5.07 ± 0.35	−0.16 ± 0.58	0.656
IL-10 (pg/mL)	8.88 (6.91–10.2)	9.13 (7.72–11.2)	0.36 (−0.66–3.24)	7.47 (6.86–8.54)	8.07 (7.58–9.57)	0.97 (−0.28–2.20)	0.981
CRP (ng/mL)	122.1 ± 47.1	130.4 ± 42.4	8.32 ± 37.3	116.7 ± 47.0	98.9 ± 51.5	−17.7 ± 46.1	0.121
BNP (pg/mL)	125 (120–155)	127 (117–136)	−4.01 (−18.7–3.07)	130 (120–136)	125 (122–130)	−3.58 (−14.2–10.3)	0.827

Values are expressed as median and 25th and 75th percentiles or as mean ± standard deviation in both groups at pre (M1) and post (M2) moments. Values are expressed as median and 25th and 75th percentiles or as mean ± standard deviation of deltas between moments M2 and M1 in both groups. TNF, tumor necrosis factor; IL, interleukin; CRP, C-reactive protein; BNP, brain natriuretic peptide. Significance level *p* < 0.05.

**Table 4:** Comparison of morphological and functional echocardiographic variables between the Control and Exercise Groups at moments M1 and M2.

Variables	Control Group (*n* = 13)			Exercise Group (*n* = 14)			*p*-Value
	M1	M2	M2-M1	M1	M2	M2-M1	
Morphological							
LVDD (mm)	50.0 ± 5.66	51.3 ± 3.99	1.31 ± 3.28	51.8 ± 6.78	52.6 ± 5.93	0.79 ± 4.26	0.726
LVSD (mm)	33.2 ± 7.68	34.5 ± 8.42	1.23 ± 2.35	30.6 ± 5.49	31.4 ± 5.98	0.79 ± 2.52	0.640
PW (mm)	8.96 ± 1.56	8.65 ± 1.30	−0.31 ± 0.81	8.69 ± 1.40	9.05 ± 1.80	0.36 ± 1.36	0.139
IVS (mm)	9.02 ± 1.76	8.81 ± 1.49	−0.22 ± 0.81	8.36 ± 1.31	9.30 ± 1.68	0.94 ± 1.58	0.027
LVMI (g/m^2^)	103.2 ± 40.0	100.4 ± 28.8	−2.81 ± 17.4	94.9 ± 22.8	106.7 ± 32.5	11.8 ± 23.8	0.082
Systolic function							
EF (%)	61.0 ± 10.1	63.0 ± 9.34	2.08 ± 4.44	66.8 ± 2.62	74.0 ± 6.40	7.21 ± 6.17	0.021
S wave (cm/s)	9.00 ± 2.27	8.61 ± 1.85	−0.39 ± 1.64	9.24 ± 1.37	9.83 ± 1.58	0.59 ± 2.18	0.203
St wave (cm/s)	14.1 ± 3.24	13.2 ± 2.64	−0.89 ± 2.58	14.1 ± 2.93	15.7 ± 3.10	1.65 ± 2.47	0.015
Diastolic function							
LAVI (mL/m^2^)	32.4 ± 9.79	32.5 ± 10.2	0.14 ± 4.85	28.0 ± 8.41	31.9 ± 11.7	3.90 ± 4.90	0.056
E wave (cm/s)	94.1 ± 24.6	92.5 ± 21.0	−1.54 ± 13.7	91.8 ± 22.8	96.1 ± 25.6	4.29± 20.6	0.398
A wave (cm/s)	53.2 ± 14.4	57.5 ± 15.9	4.31 ± 10.8	50.9 ± 17.9	53.7 ± 15.0	2.79 ± 8.80	0.691
E/A	1.82 ± 0.50	1.64 ± 0.24	−0.19 ± 0.35	1.92 ± 0.64	1.88 ± 0.62	−0.05 ± 0.52	0.422
E′ wave (cm/s)	12.0 (9.85–13.0)	11.0 (10.0–11.7)	−0.92 ± 2.37	12.0 (10.6–13.0)	13.5 (11.8–15.0)	1.51 ± 3.58	0.050
A′ wave (cm/s)	7.0 (6.0–8.0)	7.0 (7.0–8.0)	0.0 (−1.0–1.0)	8.5 (7.0–11.5)	8.0 (7.0–12.0)	0.0 (−1.0–1.0)	0.766
E/E’	8.19 ± 2.62	8.51 ± 2.03	0.32 ± 1.87	7.83 ± 2.24	7.11± 1.77	−0.72 ± 2.65	0.253
E′t wave (cm/s)	14.6 ± 3.36	12.7 ± 2.59	−1.94 ± 2.84	13.8 ± 3.31	15.0 ± 3.36	1.21 ± 4.67	0.046
A′t wave (cm/s)	9.0 (7.0–14.0)	10.0 (9.0–11.5)	0.4 (−1.0–1.5)	9.0 (7.75–15.7)	10.5 (7.0–13.8)	0.0 (−2.93–3.25)	0.788

Values are expressed as mean ± standard deviation (SD) at pre (M1) and post (M2) moments in both groups, and values are expressed as mean ± SD (standard deviation) of Diff. (differences) between M2 and M1 in the two groups. LVDD: left ventricular diastolic diameter; LVSD: left ventricular systolic diameter; PW: posterior wall diastolic thickness; IVS: interventricular septum diastolic thickness; LVMI: left ventricular mass indexed to body surface; EF: left ventricular ejection fraction; S wave: mitral annulus systolic excursion velocity on tissue Doppler (mean of medial and lateral portions); St wave: tricuspid annulus systolic excursion velocity on tissue Doppler; LAVI: left atrial volume indexed to body surface; E and A waves: peaks of mitral transvalvular flow velocity in the phase of rapid ventricular filling and during atrial contraction, respectively; E/A: ratio between E and A waves; E′ and A′ waves: velocity of diastolic excursion of the mitral annulus on tissue Doppler in the rapid filling phase and in the atrial contraction phase (average of the medial and lateral portions); Mean E/E′: ratio between E and E′ waves; E′t wave and A′t wave: velocity of diastolic excursion of the tricuspid annulus on tissue Doppler. Significance level *p* < 0.05.

**Table 5:** Comparison of physical activity level (IPAQ), functional capacity, and quality of life between the groups at M1 and M2.

Variables	Control Group (*n* = 13)			Exercise Group (*n* = 14)			*p*
	M1	M2	M2-M1	M1	M2	M2-M1	
Level of physical activity							
Job	2380 (570–4870)	2367 (764–4412)	137 (−83.5-252)	2779 (1109–5100)	2954 (1250–4477)	61.0 (−318–1111)	0.790
Transportation	641 (396–801)	785 (448–1079)	117 (45–194)	594 (211–1255)	726 (211–1539)	51.5 (0–211)	0.273
Housework	2400 (1463–2576)	2456 (1540–2655)	150 (−56–363)	1200 (485–3277)	1293 (679–2886)	105 (−180–204)	0.452
Leisure	1045 (419–2835)	1100 (624–2140)	−206.2 ± 1040	1249 (180–2400)	2803 (1897–3784)	1261 ± 860	<0.001
Walking	1300 (644–2090)	1568 (874–2240)	114 (−84.5–267)	1286 (528–2751)	2442 (805–3501)	544 (171–845)	0.024
Moderate activity	6563 ± 4584	6680 ± 4145	117 ± 1491	6769 ± 3691	7152 ± 4003	382 ± 1607	0.660
Vigorous activity	0.0 (0.0–441)	0.0 (0.0–0.0)	0.0 (−360–0.0)	0.0 (0.0–480)	0.0 (0.0–356)	0.0 (−34–113)	0.331
Activity level rating	3.0 (3.0–4.0)	3.5 (3.0–4.0)	0.0 (0.0–0.8)	4.0 (4.0–5.0)	5.0 (4.0–5.0)	1.0 (0.0–1.0)	0.111
Total activity	12,974 (9305–23,191)	14,830 (10,224–19,497)	584 (−1785–1263)	14,940 (9524–22,038)	16,155 (12,675–22,292)	1945 (957–4175)	0.021
Functional capacity							
VO_2_ peak (ml/kg/min)	35.0 (28.6–35.1)	35.0 (24.2–35.3)	0.06 (−0.55–0.57)	35.6 (35.0–47.3)	48.3 (40.2–59.9)	9.40 (1.78–16.9)	<0.001
Distance (m)	552.8 ± 160.1	586.7 ± 217.4	33.92 ± 97.2	809.1 ± 232.2	1020.7 ± 252.9	211.6 ± 130.7	<0.001
Time (min)	9.91 ± 2.13	10.3 ± 2.29	0.36 ± 0.87	12.9 ± 2.62	14.8 ± 2.13	1.88 ± 1.55	0.005
HR rest (bpm)	76.3 ± 10.6	75.9 ± 6.78	−0.39 ± 7.34	80.1 ± 14.2	72.1 ± 13.1	−8.07 ± 10.1	0.034
HR max. (bpm)	163.2 ± 12.7	166.5 ± 13.1	3.39 ± 9.73	179.1 ± 10.6	182.1 ± 12.3	3.00 ± 10.6	0.923
Quality of life							
Functional capacity	65.0 (52.5–70.0)	70.0 (61.0–82.5)	5.0 (2.5–11.5)	71.0 (63.8–80.0)	90.0 (82.5–95.0)	13.5 (6.5–20.3)	0.067
Physical aspects	60.0 (52.0–72.5)	60.0 (49.5–70.0)	0.0 (−4.0–5.0)	54.5 (25.0–100.0)	100.0 (59.3–100.0)	8.50 (0.0–41.8)	0.022
Pain	47.5 ± 20.2	54.8 ± 17.2	7.31 ± 9.38	53.4 ± 23.8	59.9 ± 29.5	6.54 ± 21.0	0.904
General state	50.2 ± 13.7	52.7 ± 15.4	2.54 ± 9.22	53.3 ± 19.7	59.6 ± 15.2	6.36 ± 18.0	0.500
Vitality	50.7 ± 10.1	51.9 ± 12.6	1.19 ± 8.00	56.8 ± 13.3	59.9 ± 12.2	3.14 ± 14.4	0.670
Social aspects	52.3 ± 21.5	52.9 ± 18.7	0.60 ± 8.01	64.9 ± 20.2	67.7 ± 19.6	2.81 ± 17.3	0.679
Emotional aspects	67.0 (52.5–80.8)	70.0 (51.3–86.0)	3.00 (−8.80–7.95)	66.8 (33.5–100.0)	100.0 (62.5–100.0)	1.5 (0.0–36.3)	0.302
Mental health	52.7 ± 18.3	52.0 ± 16.7	−0.62 ± 5.84	69.1 ± 13.7	67.6 ± 14.7	−1.43± 9.08	0.786

Values are expressed as mean ± standard deviation or median and 25th and 75% percentiles at pre (M1) and post (M2) moments in both groups, and as mean ± standard deviation or median and 25th and 75% percentiles of deltas between moments M2 and M1 in both groups. Values in MET/min/week. VO_2_ peak, oxygen consumption at peak exertion; Distance, maximum distance covered during the test; HR, heart rate (beats per minute). Significance level *p* < 0.05.

**Table 6:** Correlation of inflammatory biomarkers with time, distance walked, and peak VO_2_ of patients with sickle cell disease.

Variables	Time		Distance Walked		VO_2_ Peak	
	Correlation Coefficient	*p*-Value	Correlation Coefficient	*p*-Value	Correlation Coefficient	*p*
IL-10	−0.188	0.344	−0.220	0.266	−0.228	0.259
TNF-alpha	−0.101	0.614	−0.123	0.536	−0.130	0.524
BNP	−0.334	0.088	−0.343	0.080	−0.362	0.069
IL-1	−0.156	0.433	−0.233	0.240	−0.286	0.155
IL-6	−0.367	0.059	−0.444	0.020	−0.480	0.013

IL, interleukin; TNF, tumor necrosis factor; BNP, brain natriuretic peptide. Significance level *p* < 0.05.
